# Protective Effects of 18β-Glycyrrhetinic Acid on Monocrotaline-Induced Pulmonary Arterial Hypertension in Rats

**DOI:** 10.3389/fphar.2019.00013

**Published:** 2019-01-22

**Authors:** Min Zhang, Zhi Chang, Fang Zhao, Peng Zhang, Yin-Ju Hao, Lin Yan, Ning Liu, Jun-Li Wang, Lei Bo, Ping Ma, Wei Zhou, Xuan Ma, Qing-Bin Xu, Ru Zhou

**Affiliations:** ^1^Department of Pharmacology, College of Pharmacy, Ningxia Medical University, Yinchuan, China; ^2^General Hospital of Ningxia Medical University, Yinchuan, China; ^3^Ningxia Hui Medicine Modern Engineering Research Center and Collaborative Innovation Center, Ningxia Medical University, Yinchuan, China; ^4^Foreign Language Teaching Department, Ningxia Medical University, Yinchuan, China; ^5^Key Laboratory of Hui Ethnic Medicine Modernization, Ministry of Education, Ningxia Medical University, Yinchuan, China

**Keywords:** pulmonary arterial hypertension, monocrotaline, 18β-glycyrrhetinic acid, oxidative stress, nicotinamide adenine dinucleotide phosphate oxidase

## Abstract

Pulmonary arterial hypertension (PAH) is a destructive and rare disorder characterized by a progressive increase in pulmonary artery pressure and vasoconstriction, ultimately leading to right ventricular failure and death. 18β-Glycyrrhetinic acid (18β-GA) is an active ingredient in the commonly used Chinese herbal medicine radix glycyrrhizae, and it possesses antioxidant, anti-inflammatory, anti-tumor, and other pharmacological properties. This study aimed to determine whether 18β-GA has protective effects against monocrotaline (MCT)-induced PAH and whether it is associated with oxidative stress. The PAH of rats was induced by MCT (60 mg/kg) and oral administration of 18β-GA (100, 50, or 25 mg/kg/day), sildenafil (30 mg/kg), or saline for 21 consecutive days. The development of PAH was evaluated by hemodynamic parameters and right ventricular hypertrophy index. Hematoxylin and eosin staining, Masson trichrome staining, and electron microscopy were used to determine the degree of vascular remodeling and proliferation in lung tissue. Moreover, the antioxidant capacity and malondialdehyde levels in the lungs were measured according to the instructions provided by the test kits, and the expression levels of nicotinamide adenine dinucleotide phosphate oxidase-2 (Nox2) and Nox4 were detected through Western blot analysis. Results of our study indicated that 18β-GA treatment significantly improved the hemodynamic and pathomorphological data of the rats, reduced the changes in oxidative stress biomarkers, and inhibited Nox2 and Nox4 expression. Our research indicated that 18β-GA has a protective effect against MCT-induced PAH by inhibiting oxidative stress in rats.

## Introduction

Pulmonary arterial hypertension (PAH) is a severe and multifactorial cardiovascular syndrome that restricts flow through pulmonary arterial circulation, and it is characterized by pulmonary arterial pressure of ≥25 mmHg at rest and ≥30 mmHg when moving. It is distinguished by resistance of pulmonary arteries and an increase in pulmonary arterial pressure, which trigger subsequent right heart hypertrophy and failure and ultimately result in death (Wu et al., 2016; Gao et al., 2017; Lan et al., 2018). At present, eventful progress has been made in the comprehension of pathogenesis and treatment of PAH. However, the disease remains incurable, which is related to its relatively high morbidity and mortality. The 5- and 7-year survival rates of PAH patients are 57 and 49%, respectively (Thenappan et al., 2010; Benza et al., 2012).

The pathological mechanism of PAH is complex, involving the interaction of multiple factors. The pathogenesis of PAH includes vascular remodeling, vasoconstriction, and thrombosis, where vascular remodeling plays a critical step in the progression of PAH. Studies have reported that oxidative stress is the key mechanism leading to vascular remodeling in PAH (Demarco et al., 2010), and this condition is triggered by nicotinamide adenine dinucleotide phosphate oxidase (Nox). In PAH, Nox2, and Nox4 are up-regulated (Green et al., 2012; Wedgwood et al., 2013) and implicated in the development of increased pulmonary artery resistance and pressure (Barman et al., 2014; Wu et al., 2017a). Therefore, the antioxidation and interference of Nox may be considered therapeutic targets of PAH inhibition. Nevertheless, the conventional pharmaceutical therapies for PAH, including prostacyclin analogs, phosphodiesterase type-5 enzyme (PED-5) inhibitors, and endothelin receptor antagonists (Aiello et al., 2016), are unsatisfactory because of their toxic side effects and limited effects on the inhibition of disease progression (Humbert et al., 2010). Therefore, the discovery and development of safe and effective agents to prevent or delay the progression of PAH in the field of traditional medicine are of critical clinical significance (Galiè and Manes, 2013).

**FIGURE 1:**
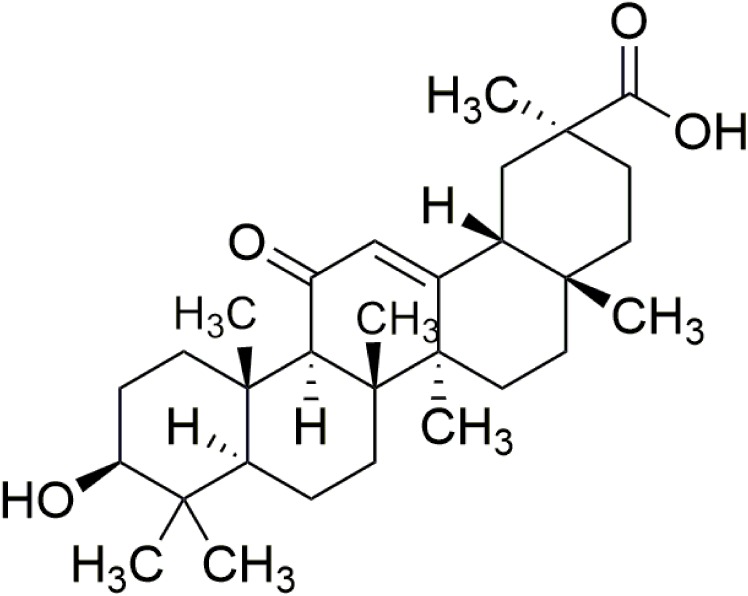
Structure of 18β-glycyrrhetinic acid. The molecular formula for 18β-glycyrrhetinic acid is C30H46O4, and the molecular weight is 470.68.

Chinese herbs have been widely investigated because of their abundant resources, safe use, and multitargeted efficacy. As a special medicinal material in Ningxia, China, radix glycyrrhizae (leguminosae) is a traditional Chinese herbal medicine that likely originated from Shennong (a specialist of herbs in ancient China) herbs. It has been widely used in the practice of traditional Chinese medicines for centuries (Chung et al., 2001; Yang et al., 2014; Young et al., 2016). Radix glycyrrhizae contains the major bioactive triterpene glycoside 18β-glycyrrhetinic acid (18β-GA), which possesses multiple pharmacological activities, such as antioxidant, anticancer, and anti-inflammation activities (Hasan et al., 2014) (Figure 1). The biological effects of 18β-GA in improving lung function have been demonstrated under various conditions in animals; for example, 18β-GA alleviates radiation-induced lung injury in mice and lung fibrosis by inhibiting inflammation (Tzuchien et al., 2010; Chen et al., 2017). 18β-GA effectively suppresses the proliferation of non-small cell lung cancer (Huang et al., 2013). All of these studies have reflected that 18β-GA has beneficial effects on lungs. However, its protective effects on PAH remain to be investigated.

On the basis of these considerations, we hypothesized that 18β-GA can improve PAH. To confirm this hypothesis, we established a PAH model of rats by single subcutaneous injection of MCT for 21 days and used 18β-GA for the therapy of rats with MCT-induced PAH (Mcmurtry et al., 2007; Wu et al., 2017a,b). Our current research aimed to examine the protective effect of 18β-GA on PAH induced by MCT and further explore its antioxidant effects.

## Materials and Methods

### Animals

Male Sprague–Dawley rats (220–260 g, 7 weeks old) were supplied by the animal experimental center of Ningxia Medical University (SYXK Ningxia 2015-0001). The animal experimental procedures were approved in accordance with the Institutional Animal Care and Use Committee of Ningxia Medical University. All the rats were housed under specific conditions (12 h light/12 h dark cycle, 22 ± 3°C) and given free access to water and food.

### Reagents

18β-GA with purity of greater than 99.3% revealed by HPLC analysis was purchased from Yuan Ye Biotechnology Co., Ltd. (Shanghai, China, Cat NO. F20O8J46129). MCT was provided by Sigma (St. Louis, MO, United States). Anti-β-actin (Cat NO. 20536-1-AP) polyclonal antibodies were provided by Proteintech Group (CA, United States). Anti-NADPH oxidase 4 antibody and anti-Nox-2/gp91phox antibody were purchased from ABCam Biotechnology (Cat NO. 14347-1-AP, 19013-1-AP). Determination kits for superoxide dismutase (SOD) (Cat NO. A001-1), malonyldialdehyde (MDA) (Cat NO. A003-1), glutathione peroxidase (GSH-PX) (Cat NO. A005), total antioxidant capacity (T-AOC) (Cat NO. A015), and catalase (CAT) (Cat NO. A007-1) were procured from Nanjing Jiancheng Research Institute of Biotechnology (Jiangsu, China).

### Experimental Design

**FIGURE 2:**
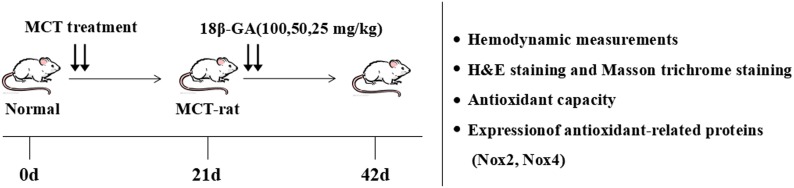
Experimental design. PAH model of rats was established by single subcutaneous injection of MCT (60 mg/kg). After 21 days, 18β-GA (100, 50, and 25 mg/kg/day) was intragastrically administered. At the end of the treatment, lung structure and function were evaluated through various experimental methods.

One hundred and twenty male rats were randomly assigned to six groups: control group (normal control, *n* = 20), model group (MCT exposure, *n* = 20), sildenafil group (MCT+sildenafil 30 mg/kg/day, *n* = 20), and 18β-GA groups (MCT+18β-GA 100, 50, and 25 mg/kg/day, *n* = 20). MCT (60 mg/kg) was administered to induce PAH by single abdominal subcutaneous injection. The control group simultaneously received normal saline on day 0. Subsequently, 18β-GA (100, 50, and 25 mg/kg/day, dissolved in saline) or sildenafil (30 mg/kg/day, suspended in saline) (Wu et al., 2017a,b) was intragastrically administered once daily from days 21 to 42. The same volume of physiological saline was given to the control and MCT-exposed groups. The Figure 2 illustrates the experimental design, including induction of PAH, subsequent administration time, and other experimental analyses.

### Survival Analysis

Survival was assessed over the entire experimental period from days 1 to 42, during which the effect of 18β-GA on survival rate of MCT-injected rats was examined. Survival curves were compared using Kaplan–Meier analysis.

### Hemodynamic Measurements

The rats were anesthetized by intraperitoneal injecting 20% urethane (1 ml/100 g), and their pressure was measured. Following stable anesthesia, the rats were placed on an operating table in a supine position. A heparin-filled polyethylene catheter was inserted into the right ventricle through the right external jugular vein to detect the mean pulmonary arterial pressure (mPAP) and right ventricular systolic pressure (RVSP) via an MPA-cardiac function acquisition analysis system (Alcott Biotech, Shanghai, China).

### Evaluation of Right Ventricular Hypertrophy

Following the pressure measurements, the rats were sacrificed, and the lungs and hearts were obtained. The lung was separated into two parts for histopathological examination and protein assay. The hearts were divided into the right ventricle (RV) and left ventricle (LV) plus the inter-ventricular septum (S). The ratio of the weight of the RV to the LV plus S [RV/(LV + S)] was calculated as the right ventricular hypertrophy index (RVHI).

### Histomorphometric Analysis

#### H&E Staining and Masson Trichrome Staining

The rats were sacrificed by cervical dislocation under anesthesia following hemodynamic measurements. The isolated lower lobe of the left lung tissue was rinsed with physiological saline and fixed with 4% paraformaldehyde for 48 h for morphometric analysis. After 48 h of fixation, the lung tissues were embedded in paraffin, cut into 4 μm-thick sections, and subjected to hematoxylin and eosin (H.E) staining and Masson trichrome staining. The structures of the pulmonary arteries and the degree of fibrosis in the artery wall in the lungs were remodeled and examined through microscopic assessment. Twenty small pulmonary vessels with diameters of 50–300 μm were randomly selected from each section and analyzed at a magnification of 400×. Two indices reflecting pulmonary arterial remodeling were calculated as follows: (1) ratio of pulmonary arterial wall thickness (WT%) = 100% × (external diameter – internal diameter)/external diameter and (2) ratio of pulmonary arterial wall area (WA%) = 100% × (transection area of the walls-lumen area)/transection area of the walls.

#### Morphological Evaluation by the Electron Microscope

After right heart catheterization of pressure measurements, a sample of the left lung tissues was collected, fixed for 2 h in Bouin’s fixative at 4°C, separated into 1 mm × 1 mm × 1 mm cubes, rinsed three times with phosphate buffer, immersed in 2% osmium tetroxide, dehydrated with an alcohol gradient, and embedded. Ultrathin 75 nm-thick sections were collected and stained with uranyl acetate and lead citrate. In the sections of lung tissues, the histopathological changes were investigated under electron microscopy (Olympus, Tokyo, Japan, 6,000×), and images were randomly obtained in a blinded manner (Karpuz et al., 2017; Shi G.J. et al., 2017; Zhang et al., 2017).

### Measurements of Oxidative Stress

All the rats were sacrificed after hemodynamic measurements. The prepared lung tissues were homogenized in ice-cold saline (0.9%) in glass homogenizer. Then the homogenate was centrifuged at 2500 r/min for 10 min at 4°C temperature. Supernatant of homogenate (10%, w/v) was employed for the subsequent tests. The activities of SOD, CAT, MDA, GSH-PX, and T-AOC in the supernatant was determined according to the instructions of the respective testing kit (Nanjing Jiancheng Bioengineering Research Institute, Jiangsu, China) and a microplate reader (1510, Thermo Fisher, United States).

### Western Blot Analysis

As described previously, the lung tissue samples from all of the groups were homogenized in lysis buffer at 4°C. The soluble proteins of Nox2 and Nox4 were collected and centrifuged at 12,000 rpm for 20 min at 4°C. The total protein concentration was measured using the BCA protein assay kit (Beyotime, Jiangsu, China). The equivalent amounts of protein lysates in each group were separated by 8% sodium dodecyl sulfate–polyacrylamide gel electrophoresis (SDS-PAGE), and the proteins on the gel were subsequently transferred onto a nitrocellulose membrane (200 mA, 2 h). The membranes were blocked with blocking PBST (PBS involving 0.1% Tween-20) buffer containing 5% skim milk at room temperature for 2 h and then probed with primary rabbit monoclonal anti-Nox-2 antibody (1:3000; ABCam Biotechnology, CA, United States) and rabbit monoclonal anti-Nox-4 antibody (1:1500; ABCam Biotechnology, CA, United States) overnight at 4°C. Subsequently, the membranes were washed with PBST and incubated with goat anti-rabbit IgG antibody (1:3000; Proteintech Group, United States) for 2 h at room temperature. The anti-β-actin monoclonal antibody (1:1000; Proteintech Group, United States) served as a control. Membranes were washed three times in PBST, and the protein bands were visualized with enhanced chemiluminescent reagents (Applygen technology, Beijing, China). The grayscale values of each band on the blots were analyzed with Quantitative One (Bio-Rad Company, CA, United States).

### Statistical Analysis

Experimental data were analyzed with SPSS software 19.0 (SPSS Inc., Chicago, IL, United States) and plotted as the mean ± standard error of the mean (SEM). Data were examined through one-way ANOVA and Student’s Newman–Keuls test for *post hoc* test to determine differences between the control and experimental groups. In all of the statistical tests, *p* < 0.05 was defined statistically significant.

## Results

### Effects of 18β-GA on the Survival of Rats

**FIGURE 3:**
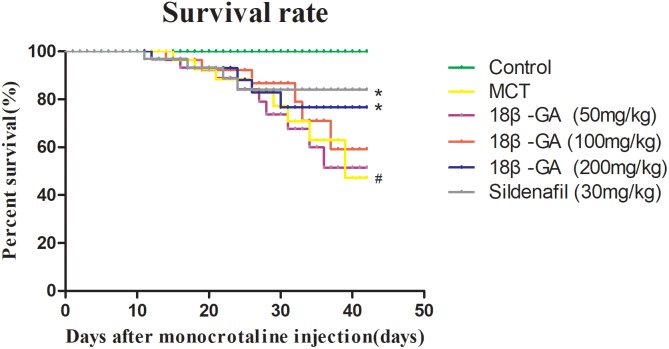
Survival analysis of all rats. Data are expressed as mean ± SEM (*n* = 20). #*p* < 0.05 vs. control group, ^∗^*p* < 0.05 vs. MCT group. MCT, monocrotaline; 18β-GA, 18β-glycyrrhetinic acid.

After MCT was injected, PAH developed and continued to cause the deterioration of the rats until their deaths. The rats’ survival rate was assessed in the entire experimental period of 42 days. The effects of 18β-GA on the survival rate of different groups of rats were also examined in the same period. At the end of our experiment, no deaths were observed in the control group, whereas the survival rates of the 18β-GA-treated group (100 mg/kg) and sildenafil group were significantly higher than that of the PAH group (*p* < 0.05; Figure 3).

### Effects of 18β-GA on Hemodynamic Measurement

**FIGURE 4:**
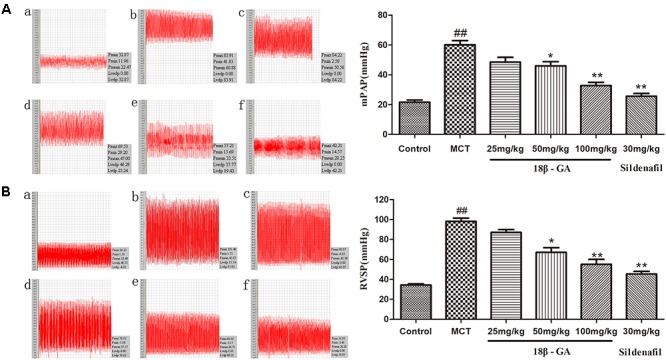
Effect of 18β-glycyrrhetinic acid treatment on MCT-induced pulmonary arterial hypertension. **(A)** Effect of 18β-glycyrrhetinic acid on mean pulmonary arterial pressure (mPAP). **(B)** Effect of 18β-glycyrrhetinic acid on right ventricular systolic pressure (RVSP). **(a)** Control group; **(b)** MCT group; **(c)** 18β-GA 25 mg/kg group; **(d)** 18β-GA 50 mg/kg group; **(e)** 18β-GA 100 mg/kg group; **(f)** Sildenafil 30 mg/kg group. Data are expressed as mean ± SEM (*n* = 10). ^##^*p* < 0.01 vs. control group, ^∗^*p* < 0.05, ^∗∗^*p* < 0.01 vs. MCT group. MCT, monocrotaline; 18β-GA, 18β-glycyrrhetinic acid; mPAP, mean pulmonary arterial pressure; RVSP, right ventricular systolic pressure.

We assessed the protective effect of 18β-GA on MCT-induced PAH by detecting the levels of mPAP and RVSP. At 6 weeks after MCT was administered, mPAP and RVSP levels significantly increased in the MCT group compared with that in the control group (*p* < 0.01; Figure 4), and this result demonstrated the successful establishment of the MCT-induced rat model. We started treatment intervention with 18β-GA and sildenafil at 3 weeks after MCT exposure. The rats treated with 18β-GA (100 and 50 mg/kg) and sildenafil (30 mg/kg) demonstrated significantly lower mPAP and RVSP levels than the MCT group (*p* < 0.01 and *p* < 0.05, respectively; Figure 4). All these results demonstrated that oral administration of 18β-GA could alleviate MCT-induced PAH in rats.

### Effects of 18β-GA on Right Heart Hypertrophy

**FIGURE 5:**
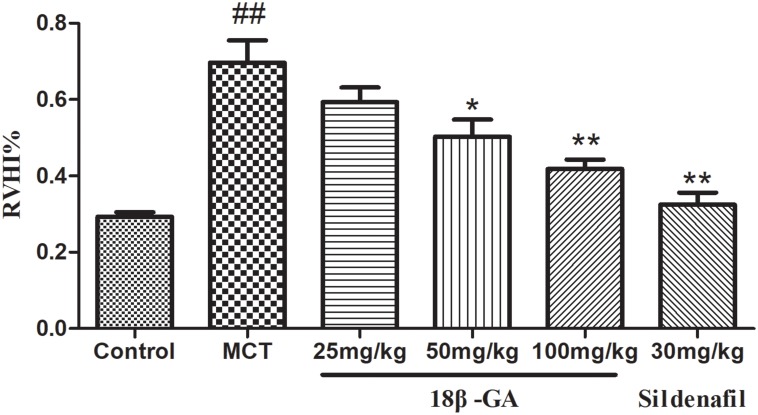
Effect of 18β-glycyrrhetinic acid on right ventricular hypertrophy index (RVHI). Data are expressed as mean ± SEM (*n* = 10). ^##^*p* < 0.01 vs. control group, ^∗^*p* < 0.05, ^∗∗^*p* < 0.01 vs. MCT group. MCT, monocrotaline; 18β-GA, 18β-glycyrrhetinic acid; RVHI, right ventricular hypertrophy index.

The weight ratio of RV/LV+S was calculated to assess the extent of right ventricular hypertrophy (RVH). A significant increase in RVHI was observed due to an increase in pulmonary arterial pressure. Changes in this ratio further confirmed that severe PAH was induced after MCT was subcutaneously injected. In the MCT-treated group, RVHI significantly increased (*p* < 0.01 and *p* < 0.05; Figure 5). In comparison with RVHI in the MCT group, RVHI could be markedly decreased by two doses of 18β-GA (100 and 50 mg/kg) and sildenafil (30 mg/kg) (*p* < 0.01 and *p* < 0.05, respectively; Figure 5). As expected, 18β-GA obviously alleviated MCT-induced RVH.

### Effects of 18β-GA on Pulmonary Vascular Remodeling in MCT Induced PAH

#### H&E Staining and Masson Trichrome Staining

**FIGURE 6:**
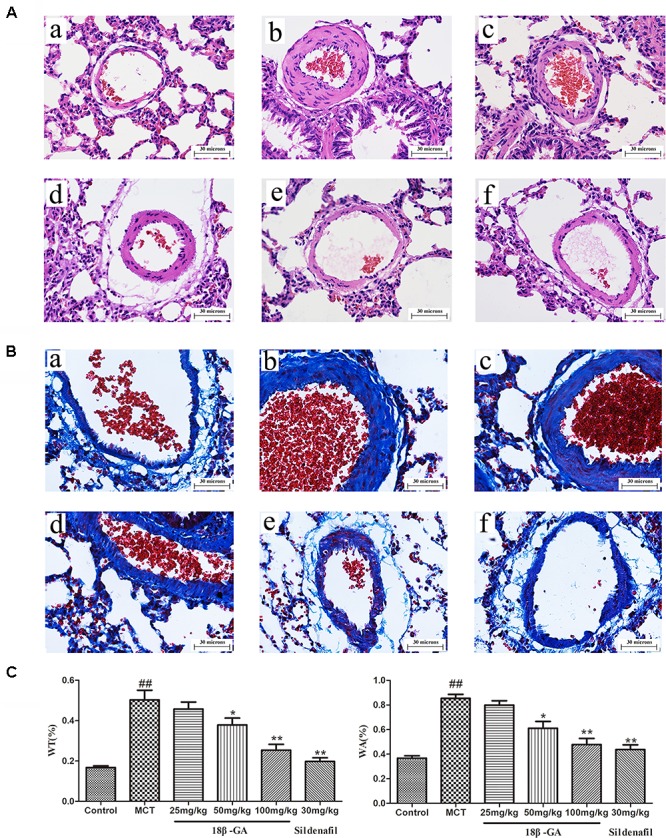
Effect of 18β-glycyrrhetinic acid on MCT-induced pulmonary vascular remodeling. Representative photomicrographs of pulmonary small arteries by H&E staining **(A)** and Masson staining **(B)** (magnification 400×): **(a)** control group; **(b)** MCT group; **(c)** 18β-GA 25 mg/kg group; **(d)** 18β-GA 50 mg/kg group; **(e)** 18β-GA 100 mg/kg group; **(f)** Sildenafil 30 mg/kg group. **(C)** Left: Effect of 18β-GA on WT%, Right: Effect of 18β-GA on WA%. Data are expressed as mean ± SEM (*n* = 10). ^##^*p* < 0.01 vs. control group, ^∗^*p* < 0.05, ^∗∗^*p* < 0.01 vs. MCT group. MCT, monocrotaline; 18β-GA, 18β-glycyrrhetinic acid; WT%, the ratio of the vascular walls thickness; WA%, the ratio of the vascular wall area.

Pulmonary vascular remodeling is one of the crucial causes leading to PAH development. We performed hematoxylin and eosin (H&E) staining and Masson trichrome staining to evaluate the pathological changes in the small pulmonary arteries (50–200 μm). H&E staining demonstrated that the pulmonary artery of the control group was characterized by a thin-walled medial wall and a large lumen (Figure 6A). In comparison with the lung histology of the control group, the MCT group demonstrated increased pulmonary arterial wall thickness and luminal stenosis, as well as excessive inflammatory cell count. After the rats were treated with 18β-GA and sildenafil, the changes in pulmonary arterial morphology significantly improved. In addition, pulmonary parietal wall thickness indices (WA% and WT%) were determined to assess the effect of 18β-GA on pulmonary vascular remodeling. WA% and WT% remarkably increased in the MCT-treated group compared with those in the control group (*p* < 0.01 and *p* < 0.05; Figure 6A). In the 18β-GA (100 and pmg mg/kg) and sildenafil (30 mg/kg) groups, WA% and WT% decreased compared with the MCT group (*p* < 0.01 and *p* < 0.05, respectively; Figure 6C). In the control group, Masson staining displayed a small amount of collagen fibers in the field of vision (Figure 6B), but the MCT group demonstrated many disordered proliferating collagen fibers in the blood vessel wall and surrounding tissues (Figure 6B). Similar to the sildenafil-treated rats, 18β-GA-treated rats showed a significant improvement in collagen fiber proliferation (Figure 6B). These results indicated that 18β-GA administration significantly improved the pathological changes in the pulmonary vasculature induced by MCT.

#### Electron Microscopy Analyses

**FIGURE 7:**
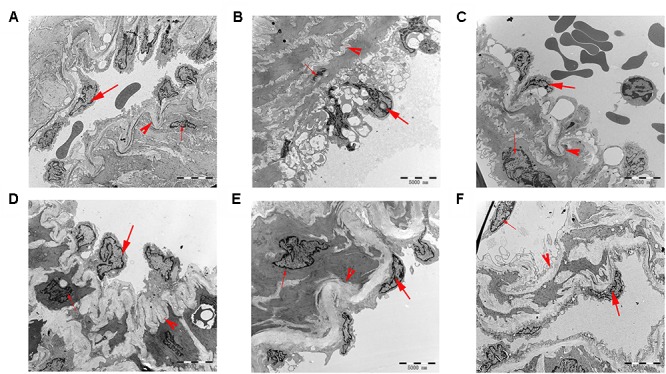
Ultrathin cross-sections of the pulmonary vascular in rats were examined under transmission electron microscopy (6,000× magnification; bar = 5,000 nm). **(A)** Control group; **(B)** MCT group; **(C)** 18β-GA 25 mg/kg group; **(D)** 18β-GA 50 mg/kg group; **(E)** 18β-GA 100 mg/kg group; **(F)** Sildenafil 30 mg/kg group. Endothelial cell (thick arrow), smooth muscle cell (thin arrow), and elastic membrane (arrow head). MCT, monocrotaline; 18β-GA, 18β-glycyrrhetinic acid.

Changes in pulmonary artery ultrastructure were analyzed by transmission electron microscopy. Under the electron microscope, the pulmonary vascular of the rats in the control group displayed normal morphological characteristics. Endothelial cells, smooth muscle cells, and connective tissue components were also detected as normal. In the MCT group, the endothelial cells in the middle and small muscles of the lungs were hyperplastic, hypertrophic, and swollen; the smooth muscle cells were hypertrophic. The elastic membrane structure was incomplete and fractured. A large number of mitochondria and endoplasmic reticuli were observed in the cytoplasm. After treatment with 18β-GA and sildenafil, the endothelial cells of the middle and small muscular arteries in the rats were flat, the vacuoles in the cytoplasm significantly decreased, the elastic membrane structure was clear, and the ultrastructural changes in the pulmonary artery significantly improved compared with those in the model group. These results suggested that the histopathological features of the lung tissues of the rats in the 18β-GA-treated groups were alleviated (Figure 7).

#### Effects of 18β-GA on Oxidative Stress

**FIGURE 8:**
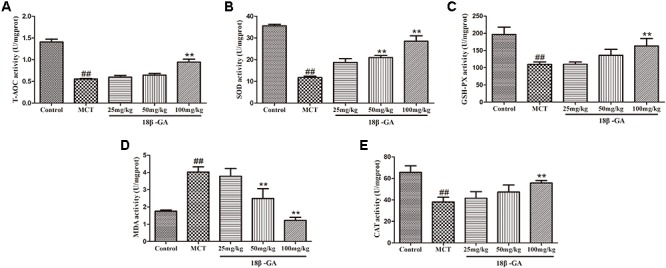
Effect of 18β-glycyrrhetinic acid on oxidative stress induced by MCT. **(A)** Effect of 18β-glycyrrhetinic acid on T-AOC expression. **(B)** Effect of 18β-glycyrrhetinic acid on SOD expression. **(C)** Effect of 18β-glycyrrhetinic acid on GSH-PX expression. **(D)** Effect of 18β-glycyrrhetinic acid on MDA expression. **(E)** Effect of 18β-glycyrrhetinic acid on CAT expression. Data are expressed as mean ± SEM (*n* = 10). ^##^*p* < 0.01 vs. control group, ^∗∗^*p* < 0.01 vs. MCT group. T-AOC, total antioxidant capacity; SOD, determine superoxide dismutase; GSH-PX, glutathione peroxidase; MDA, malonyldialdehyde; CAT, catalase; MCT, monocrotaline; 18β-GA, 18β-glycyrrhetinic acid.

The biomarker levels of oxidative stress in lung tissues were measured to demonstrate the effects of 18β-GA on MCT-induced oxidative stress. In comparison with the levels in the control group, the SOD, CAT, T-AOC, and GSH-PX levels in the MCT-exposed group markedly decreased, whereas their MDA levels significantly increased (*p* < 0.01 and *p* < 0.05, respectively; Figure 8). All these results implied that MCT could aggravate oxidative stress. Subsequently, administration of 18β-GA down-regulated the MDA level and increased SOD, CAT, T-AOC, and GSH-PX activity (*p* < 0.01 and *p* < 0.05, respectively; Figure 8). Therefore, 18β-GA treatment inhibited MCT-induced oxidative stress.

### Effects of 18β-GA on Nox2 and Nox4 Expression

**FIGURE 9:**
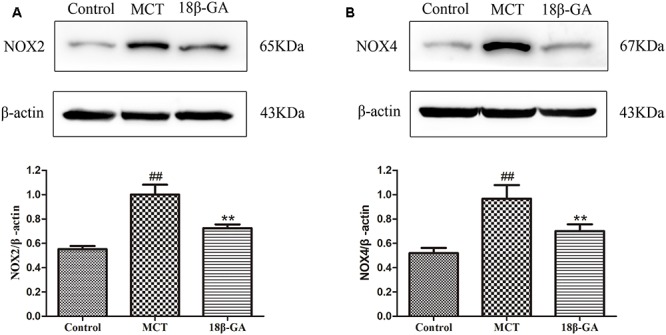
Effects of 18β-glycyrrhetinic acid on NOX-2 and NOX-4 expression. **(A)** Representative Western blot band of NOX-2 activation in the lung tissues. **(B)** Representative Western blot band of NOX-4 activation in the lung tissues. Data are expressed as mean ± SEM (*n* = 10). ^##^*p* < 0.01 vs. control group, ^∗^*p* < 0.05, ^∗∗^*p* < 0.01 vs. MCT group. NOX-2, nicotinamide adenine dinucleotide phosphate (NADPH) oxidase-2; NOX-4, nicotinamide adenine dinucleotide phosphate (NADPH) oxidase-4; MCT, monocrotaline; GA, 18β-glycyrrhetinic acid.

Based on the above studies, we found that 18β-GA can counteract the MCT-induced oxidation. To further investigate the link between 18β-GA and antioxidant effects, we examined the influence of 18β-GA on the protein expression of Nox2 and Nox4, which could be related to antioxidant effects. The results indicated that the protein expression levels of Nox2 and Nox4 were up-regulated in the MCT-treated group compared with those in the control group (*p* < 0.01 and *p* < 0.05, Figure 9, respectively). 18β-GA (100 mg/kg) treatment could reverse the increase in the Nox2 and Nox4 expression levels in the lung tissue of the model rats, but their levels remained higher than those of the control group (*p* < 0.01 and *p* < 0.05, Figure 9, respectively). These results suggested that 18β-GA may against the progression of PAH at least partly via inhibition of the protein expression of Nox2 and Nox4.

## Discussion

This study reported for the first time that the intragastric administration of 18β-GA for 21 consecutive days exerted protective effects against MCT-induced PAH in rats. The present study demonstrated that the survival rate of PAH model rats treated with 18β-GA significantly improved. We observed that treatment with 18β-GA attenuated lung and heart damage and inhibited the increase in mPAP, RVSP, and RVHI levels in the MCT-induced rat model. Histological verification showed that the pathological changes in the pulmonary vasculature induced by MCT were reversed. WA% and WT% of the pulmonary artery improved, whereas the proliferation of pulmonary fibrous tissue decreased. Meanwhile, oxidative stress was inhibited in the rats with MCT-induced PAH. Western blot analysis results revealed that 18β-GA administration also down-regulated the protein overexpression of Nox2 and Nox4 in the lungs. In summary, 18β-GA provided a protective effect against MCT-induced PAH by inhibiting oxidative stress.

Pulmonary arterial hypertension, a pathological condition with low survival rates, is characterized by an increase in pulmonary arterial pressure, excessive pulmonary vascular remodeling, and subsequent right heart dysfunction (Haga et al., 2014; Lee et al., 2016). In this experiment, we used the rat model induced by MCT. When MCT was administered, its metabolites were distributed via pulmonary circulation in the body, where they could damage pulmonary vascular endothelial cells and cause vascular stenosis. The resistance in lung vasculature, pulmonary arterial wall remodeling, and right ventricular hypertrophy increased. Consequently, PAH developed. This model can mimic many characteristics of human PAH (Gomezarroyo et al., 2012; Wu et al., 2017b). The MCT-induced PAH model is also characterized by elevated levels of superoxide in the lungs and right ventricles (Singal et al., 2004; Kamezaki et al., 2008; Demarco et al., 2010). Thus, we chose the MCT-induced PAH model for our research.

18β-GA is a biologically active ingredient of licorice with an evident antioxidant effect and promising lung protective activity (Tzuchien et al., 2010; Huang et al., 2013; Hasan et al., 2014; Chen et al., 2017). In our pre-experiment, the optimal dosage range was 25–100 mg/kg (Oztanir et al., 2014; Mahmoud et al., 2017), and no significant toxicity was observed. On the basis of treatment strategies associated with clinical practices and our previous research experience, we used 18β-GA after MCT was subcutaneously injected for 21 days and observed the survival rate of rats during the administration period (Mcmurtry et al., 2007; Wu et al., 2017a,b). A decline in the survival rate of PAH rat models was used as a marker of disease deterioration (Sun et al., 2017). In our experiment, the survival rate of 18β-GA-treated rats significantly improved compared with that in the MCT group. Therefore, 18β-GA could improve the quality of life of animals suffering from PAH.

Human pulmonary hypertension is characterized by abnormal pulmonary hemodynamic changes, including a marked increase in mPAP and RVSP and occurrence of RVH (Temple et al., 2014; Brewis et al., 2015). In animal models, the similar changes in mPAP, RVSP, and RVHI are often considered evidence of PAH development (Shi R. et al., 2017). Therefore, our study examined the effects of 18β-GA on hemodynamic parameters and RVHI. Our results suggested that 18β-GA therapy blocked the increase in mPAP, RVSP, and RVHI in a dose-dependent manner. These findings indicated that 18β-GA could prevent PAH.

Abnormal pulmonary vascular remodeling is considered the primary pathological feature of PAH (Bai et al., 2017), including pulmonary artery wall thickening and pulmonary artery stenosis. These changes can cause vasoconstriction resistance and increased cardiac after-load, eventually leading to PAH. H&E staining and Masson trichrome staining were applied to measure the medial wall thickness of the pulmonary artery (WA% and WT%) and degree of pulmonary fibrosis and evaluate pulmonary vascular remodeling (Guignabert et al., 2013; Aiello et al., 2016; Lee et al., 2016). Transmission electron microscopy was performed to analyze the changes in the ultrastructure of the pulmonary artery. The pulmonary vascular medial wall thickness and vascular proliferation significantly increased in the MCT-treated groups, and this increase was also an indicator of PAH. Under the electron microscope, the smooth muscles of the pulmonary arterioles were hyperplastic, the collagen fibers of the adventitia were dense, and the endothelial cells were prominent in the model group. These features were effectively changed by the daily intragastric administration of 18β-GA, suggesting that 18β-GA elicited a protective effect against PAH by curbing pulmonary vascular remodeling.

Oxidative stress is a critical contributory element in various physiological and pathological processes and in the pathogenesis of PAH caused by MCT (Demarco et al., 2010). The pathological features of PAH mainly include pulmonary vasoconstriction and pulmonary artery wall remodeling (Bai et al., 2017). Oxidative stress has been described in the literature as a pathogenic mechanism of vascular remodeling observed in PAH (Barman et al., 2014). In the body, most of the vascular cells, including endothelial cells, smooth muscle cells, and adventitial cells, produce reactive oxygen species. Among the patients with vascular disease, the oxidative–antioxidative balance in blood vessel walls is impaired due to the increased reactive oxygen produced by these cells, thereby promoting vasoconstriction, smooth muscle cell proliferation, and vascular remodeling (Zhang et al., 2015). These findings suggested that limiting oxidative stress could help alleviate PAH. After reviewing many references, we found that oxidative stress is characterized by an increase in the production of oxidants (e.g., MDA) and a decrease in the concentrations of antioxidants and antioxidant enzymes (e.g., SOD, CAT, and GSH-PX) (Zimmermann et al., 2004). MDA is a toxic product of lipid peroxidation and a sensitive marker of oxidative stress (Chen et al., 2011). High-performance antioxidant defense systems, including endogenous antioxidant enzymes (such as GSH-PX, SOD, CAT, and T-AOC), play an critical role in maintaining low oxidant concentrations and redox balance and in determining the active state that can reflect the degree of oxidative stress (Tabima et al., 2012). In our study, we examined the changes in SOD, CAT, MDA, T-AOC, and GSH-PX levels. Compared with the control group, MCT-treated rats presented significantly reduced SOD, CAT, T-AOC, and GSH-PX concentrations and increased MDA levels, but 18β-GA administration reversed these changes.

Nox-derived products can trigger oxidative stress. In the vasculature, they can help maintain vascular tone and regulate important processes, such as cell growth, proliferation, differentiation, apoptosis, cell migration, and cytoskeletal organization (Wolin et al., 2005; Manea, 2010). The Nox family is composed of seven members; among them, Nox2 and Nox4 are the chief isomers distributed in the cardiovascular system. Nox2 and Nox4 are up-regulated in lung tissues from animal models and humans with PAH (Green et al., 2012; Barman et al., 2014), and they are likely involved in the development of increased pulmonary artery resistance and pressure (Liu et al., 2006; Demarco et al., 2010; Wu et al., 2017a). Nox2 plays a significant role in endothelial dysfunction in PAH (Li et al., 2008; Liu et al., 2016). Nox4 is up-regulated in rats with MCT-treated and chronic hypoxia-induced PAH (Nisbet et al., 2009). Nox4 promotes the hypoxia-induced growth of HPASMCs, and silencing of Nox4 expression by RNA interference can reduce the proliferation of human PASMCs and fibroblasts (Sanders and Hoidal, 2007). In this study, we investigated the protein expression of Nox2 and Nox4 by Western blot analysis. The results showed that the protein levels of Nox2 and Nox4 in the 18β-GA-treated group were obviously lower than those in the MCT-treated group. These experimental results indicated that 18β-GA could attenuate oxidative stress and reduce Nox2 and Nox4 expression.

According to the control principle of the experimental program, the positive drug group is necessary to verify the rationality of our experimental program and the reliability of the protective effects of 18β-GA against PAH. Several studies have reported that sildenafil possesses a protective effect on MCT-induced PAH (Jasińskastroschein et al., 2014). The Food and Drug Administration also certified sildenafil for use in the treatment of PAH. Therefore, sildenafil was selected as a positive agent, and it exerted a protective effect on MCT-induced PAH as previously described in our study. There are some limitations in this study. The pathological mechanism of PAH is complex, such as and inflammation endothelial dysfunction. However, we have only studied the antioxidant effects of 18β-GA on PAH, which is not comprehensive and in-depth. Therefore, we will thoroughly test the effects of 18β-GA on the inflammatory process in future studies.

## Conclusion

In this study, we first demonstrated that 18β-GA administered to rats with PAH induced by MCT elicited a significant protective effect. 18β-GA treatment improved hemodynamics and right ventricular hypertrophy and reduced cardiopulmonary injury. The protective effect of 18β-GA against PAH was related with the inhibition of oxidative stress and down-regulation of Nox2 and Nox4 levels. Therefore, the experimental data supported the view that 18β-GA is beneficial for the treatment of PAH.

## Ethics Statement

This study was carried out in accordance with the recommendations of the Institutional Animal Care and Use Committee of Ningxia Medical University. The protocol was approved by the Institutional Animal Care and Use Committee of Ningxia Medical University.

## Author Contributions

ZC, NL, J-LW, and Y-JH performed data curation. RZ and Q-BX conducted project administration. LY, PM, WZ, LB, and XM carried through supervision. MZ processed writing-original draft. RZ and MZ executed writing-review and editing. All authors commented on and approved the final manuscript.

## Conflict of Interest Statement

The authors declare that the research was conducted in the absence of any commercial or financial relationships that could be construed as a potential conflict of interest.
